# Patterns of Engagement With an AI Conversational Agent for Mental Health and Associations With Anxiety and Depression: Cross-Sectional Study

**DOI:** 10.2196/98690

**Published:** 2026-08-10

**Authors:** Kelsey McAlister, Courtney Jewell, Jennifer Huberty

**Affiliations:** 1 Fit Minded, Inc, 2901 E Greenway Rd PO Box 30271, Phoenix, AZ, 85032, United States, 1 602 935-6986

**Keywords:** user engagement, digital mental health, AI-powered interventions, real-world data, digital therapeutics

## Abstract

**Background:**

Digital mental health interventions using conversational AI agents are increasingly being adopted as scalable alternatives to traditional care. Engagement is typically measured using volume-based metrics (eg, session counts and total time on a platform). However, these metrics overlook engagement patterns over time, which are not well understood in relation to mental health outcomes.

**Objective:**

The purpose of this cross-sectional study was to explore how different patterns of engagement with Mental’s AI conversational agent relate to self-reported depression and anxiety. We aimed to (1) identify and describe engagement profiles based on patterns of interaction depth and temporal consistency, (2) compare depression and anxiety symptoms across engagement profiles, and (3) explore whether engagement profiles were associated with mental health symptom severity.

**Methods:**

This cross-sectional observational study linked survey responses to back-end app usage data from 112 Mental app users who completed at least 5 sessions with the conversational AI agent. Engagement profiles were derived using median splits on interaction depth (α parameter) and temporal consistency (Gini coefficient). Depression was assessed using the Patient Health Questionnaire-8 (PHQ-8), and anxiety was assessed using the Generalized Anxiety Disorder-7 (GAD-7). One-way ANOVAs compared symptoms across profiles. Linear regression models examined associations between profiles and symptom severity, adjusting for age, gender, and total duration of use.

**Results:**

We identified 4 distinct engagement profiles based on interaction depth and temporal consistency: extended and episodic (profile 1; n=25), extended and consistent (profile 2; n=31), brief and episodic (profile 3; n=31), and brief and consistent (profile 4; n=25). Users in profile 1 (extended and episodic) reported the lowest anxiety (mean 2.68, SD 2.43) and depression (mean 3.48, SD 4.06), while profile 4 (brief and consistent) reported the highest anxiety (mean 10.00, SD 7.03) and depression (mean 10.60, SD 8.75). Significant differences were observed for anxiety (*F*_3,108_=8.07, *P*<.001, *η*²=0.18) and depression (*F*_3,108_=5.47, *P*=.002, *η*²=0.13). In adjusted models, engagement profile was significantly associated with depression (*R*²=0.16, *F*_7,104_=2.87, and *P*=.009) and anxiety (*R*²=0.21, *F*_7,104_=4.04, and *P*<.001). Compared to profile 1, users in profiles 2 and 4 reported significantly higher depression and anxiety. Profile 3 differed from profile 1 for anxiety only (β=3.11, *P*=.047).

**Conclusions:**

Users with longer, clustered sessions reported the lowest symptoms, whereas those with brief, evenly distributed use reported the highest symptom levels, suggesting that the structure of engagement may be associated with symptom levels in ways that aggregate usage metrics do not capture. These findings are preliminary and hypothesis-generating, highlighting the importance of considering how engagement unfolds over time and suggesting that pattern-based measurement may improve the understanding of user outcomes in AI-powered mental health care. Future work should examine the directionality of these associations and whether distinct engagement patterns reflect meaningfully different modes of interacting with AI-powered care.

## Introduction

Mental health disorders affect an estimated 1 in 8 people globally, yet most who need care never receive it [1]. In the United States, almost half of individuals with mental illness do not receive care, with gaps driven by cost, geographic access, and stigma [2,3]. These shortfalls have accelerated interest in digital mental health interventions (DMHIs) as scalable, accessible alternatives to traditional care [4]. Among DMHIs, conversational AI agents, commonly referred to as AI therapists or mental health chatbots, have emerged as a particularly promising modality, offering around-the-clock availability and low cost [4]. Conversational AI agents can produce meaningful reductions in symptoms of depression and anxiety [5,6], lending empirical credibility to their growing adoption and positioning them as a practical option in settings where traditional care is hard to access.

Despite the promise of conversational AI agents, a critical gap remains in understanding what drives therapeutic benefit within AI-powered care. Engagement in DMHIs is most commonly operationalized as the number of modules, activities, or sessions completed [7], largely because these metrics are easy to extract and compare across users. However, these metrics assume that all engagement is equivalent, collapsing meaningful variation in how interactions unfold over time into a single quantity. Higher engagement does not consistently predict better outcomes in DMHIs, with the relationships between volume-based engagement and symptom improvement being weak and inconsistent [8,9]. Characterizing engagement in DMHIs has proven to be difficult, partially because engagement is multidimensional and encompasses behavioral, cognitive, and affective components that volume-based metrics cannot distinguish [10]. Prior work in DMHIs has largely focused on adherence, dropout, or total use [7,9]. As a result, existing approaches fail to capture how engagement unfolds over time, including patterns of depth and timing that may be critical for understanding outcomes.

In contrast, human psychotherapy research shows that not only the amount of care but also how it is structured over time, including session intensity and spacing, can influence clinical improvement beyond the total therapy received [11,12]. For example, twice-weekly cognitive behavioral therapy (CBT) sessions have been shown to produce faster rates of symptom improvement in depression compared to once-weekly delivery, independent of the total sessions attended, suggesting that session concentration matters beyond cumulative dose [13]. These findings suggest that engagement is not interchangeable across time and that when and how interactions occur may shape therapeutic benefits independent of overall exposure. This distinction is particularly relevant for conversational AI interventions, where engagement is highly flexible and user-driven, leading to substantial variability in how individuals interact with care over time. However, despite this variability, the temporal and structural patterns of engagement have not been systematically examined in AI-powered DMHIs, leaving a significant gap in understanding which forms of engagement are associated with better outcomes and how AI-powered platforms should be designed and evaluated.

Mental is a commercially available DMHI designed primarily for adult men. Its core feature is a generative AI conversational agent that delivers on-demand, free-text mental health support grounded in evidence-based practice. Unlike earlier DMHIs that relied on structured, rule-based, or decision-tree frameworks grounded primarily in manualized CBT [14,15], Mental’s conversational AI agent draws flexibly from multiple therapeutic frameworks, including CBT, acceptance and commitment therapy, and motivational interviewing, selecting appropriate techniques based on user input and conversational context. This approach mirrors the adaptive, individualized nature of routine clinical practice. At the start of each session, users select a preferred AI persona (eg, differing in tone, priorities, and inspirations) and a session length (approximately 8, 15, or 30 min), allowing for flexible, customizable engagement. Users then interact with the conversational AI agent through real-time, free-text dialogue. The flexible, user-driven nature of the Mental platform makes it well-suited for examining how engagement patterns vary in real-world use and whether those patterns are meaningfully associated with mental health outcomes.

The purpose of this cross-sectional study was to explore how different patterns of engagement with the Mental conversational AI agent relate to self-reported depression and anxiety. We aimed to (1) identify and describe engagement profiles based on patterns of interaction depth and temporal consistency, (2) compare depression and anxiety symptoms across engagement profiles, and (3) explore whether engagement profiles were associated with depression and anxiety symptom severity.

## Methods

### Study Design and Participants

This cross-sectional observational study analyzed survey and app-use data from the Mental app, a commercially available, evidence-based DMHI that uses a generative AI conversational agent to support users’ mental health. Participants were eligible if they met the following criteria: (1) aged ≥18 years, (2) US residents, (3) current or prior users of the Mental app’s generative AI conversational agent, (4) had completed ≥5 sessions with the conversational AI agent (ie, the minimum required for stable estimation of α and Gini coefficients), and (5) were able to read and understand English. The survey was an open, web-based survey distributed via email and was not password-protected. Eligible users were identified using back-end data and were invited via email (up to 3 times) to complete an optional survey assessing the app’s impact on various aspects of mental health, workplace productivity, and daily functioning, as well as trust, safety, and comfort with the conversational AI agent. Mental app users who completed the survey received a US $20 gift card, which was offered by the company, independent of the research protocol. Survey completion was optional.

A total of 209 survey responses were received. After removing duplicate entries based on duplicate user IDs (n=4), 205 unique users completed the survey. Survey responses were successfully matched with back-end user data for all 205 users. This study was reported in accordance with the STROBE (Strengthening the Reporting of Observational Studies in Epidemiology; Checklist 1) guidelines for observational studies and the CHERRIES (Checklist for Reporting Results of Internet E-Surveys; Checklist 2) guidelines for web-based surveys.

### Ethical Considerations

The study protocol was reviewed by the Biomedical Research Alliance of New York (BRANY) Institutional Review Board (study ID: 26-006-1708) and was determined to qualify for exemption under US federal regulations for human participants’ research involving secondary analysis of existing data [16]. Because the study used retrospective, deidentified data and involved no direct participant contact, informed consent procedures were not required. All data were deidentified before analysis. Back-end engagement data were extracted from the platform’s operational data infrastructure as part of routine app functionality, and survey data were collected as part of routine business operations. Users acknowledged the data collection practices through the Mental app’s terms of service and privacy policy at the time of account creation.

### Mental App

Mental is a commercially available DMHI developed by The Path and is available for download on iOS and Android [17]. The platform is marketed primarily to adult men and offers multiple tools, including motivational content and structured training activities, though the present study focuses specifically on user engagement with Mental’s generative AI conversational agent. The generative AI conversational agent is grounded in multiple empirically supported therapeutic approaches, including CBT, acceptance and commitment therapy, motivational interviewing, and other cognitive, behavioral, and skills-based strategies. Rather than following fixed dialogue pathways, Mental’s conversational AI agent draws flexibly from these frameworks based on user input and conversational context. At the start of each session, users select a preferred AI persona and a session length (approximately 8, 15, or 30 min), allowing flexible, customizable engagement, after which they interact with the agent through real-time, free-text dialogue.

### Study Measures

The cross-sectional survey was administered between February 16, 2026, and March 18, 2026. User engagement data, including session length and number of sessions, were extracted from October 22, 2024, to March 12, 2026, and linked to participant survey responses to explore relationships between survey responses and app engagement. The observation window of back-end data reflects the full period of available platform data at the time of the study. The survey was delivered using the web-based platform SurveyMonkey, and completion required approximately 8 minutes.

The survey was administered as part of routine business operations. It was developed by 2 doctoral-level researchers with expertise in behavior change and digital health outcomes measurement and was designed to assess clinically relevant constructs using validated instruments where available. The survey questions included validated outcome measures and investigator-developed items designed to assess users’ experiences with and perceived impact of Mental’s AI therapist. All survey questions were optional, and participants could edit their responses using a Back button. Survey items were presented in a fixed order. Participants completed 83 to 89 items across 12 to 13 pages, depending on branching logic, with 1 to 15 items per page. The survey items assessed (1) self-reported use of Mental’s AI therapist (frequency, reasons for use, and discontinuation; 3‐6 items), (2) perceived impact on mental health and emotional well-being (15 items), (3) impact on work productivity (5 items), (4) impact on health care utilization (4 items), (5) perceptions of AI safety, trust, and usability (8 items), (6) comparisons with other forms of mental health support (1‐8 items), (7) validated outcome measures (29 items), (8) demographic characteristics (4 items), and optional follow-up items (2‐4 items, including incentive and interview sign-up). Only survey items relevant to the primary research questions were analyzed and reported in this study. The complete survey instrument is provided in Multimedia Appendix 1.

Depression was assessed using the Patient Health Questionnaire-8 (PHQ-8; [18]), an 8-item self-report measure of depressive symptom severity. Respondents were asked, “Over the last 2 weeks, how often have you been bothered by any of the following problems?” Responses to these prompts were rated on a 4-point Likert scale ranging from 0 (not at all) to 3 (nearly every day), with total scores calculated as the sum of all items, ranging from 0 to 24. Higher PHQ-8 scores indicate greater depressive symptom severity. The PHQ-8 has demonstrated strong internal consistency (α=.86-.89; [18]).

Anxiety was assessed using the Generalized Anxiety Disorder scale (GAD-7; [19]), a 7-item self-report measure of generalized anxiety severity. Respondents were asked, “Over the last 2 weeks, how often have you been bothered by the following problems?” Responses to these prompts were rated on a 4-point Likert scale ranging from 0 (not at all) to 3 (nearly every day), with total scores calculated as the sum of all items, ranging from 0 to 21. Higher GAD-7 scores indicate greater anxiety symptom severity. The GAD-7 has demonstrated strong internal consistency (α=.89-.92; [19]).

### App Engagement Patterns

To address existing limitations of volume-based metrics (eg, session counts and module completions), the present study explored 2 engagement metrics to characterize how the distribution and concentration of sessions varied across users: interaction depth and temporal consistency. The α parameter, estimated from the distribution of session durations using a Pareto-based maximum likelihood approach, characterizes the shape of the session duration distribution rather than its mean, making it sensitive to variation in how deeply users engage across sessions rather than simply how much total time they accumulate [20]. Session durations across the analytic sample were right-skewed (median 14.8, IQR 11.9-22.3 min; mean 16.7, SD 57.1 min), consistent with a heavy-tailed distribution and supportive of the Pareto model as an appropriate characterization of session duration behavior. The α parameter was estimated using maximum likelihood estimation. Given that session durations were drawn from a discrete set of platform-defined lengths (approximately 8, 15, or 30 min), formal goodness-of-fit testing using the Kolmogorov-Smirnov statistic was not appropriate due to violations of the continuity assumption. Visual inspection of the log-log complementary cumulative distribution function confirmed approximately linear decay consistent with the heavy-tailed distributional assumptions of the Pareto model, supporting its use for characterizing session depth in this sample. Lower α values indicate longer, more sustained sessions, while higher α values indicate shorter, more fragmented interactions. The Gini coefficient, derived from the distribution of usage across days, quantifies inequality in the temporal spacing of engagement, with values approaching 1 reflecting highly clustered use and values approaching 0 reflecting evenly distributed use [21]. Together, these metrics map onto the 2 dimensions absent from traditional volume-based measurement: how deeply users interact within sessions and how that engagement is distributed over time [21,22]. The total number of sessions and total duration of use were also extracted from back-end usage data to provide descriptive summaries of overall app exposure.

### Statistical Analyses

Before analysis, all data were reviewed for completeness and internal consistency. Duplicate entries were identified and removed based on unique user IDs, and back-end engagement records were cross-checked against survey responses to confirm successful linkage across all retained cases. Given the retrospective, exploratory nature of this study, an a priori power analysis was not conducted. Sessions exceeding 240 minutes were identified as likely data artifacts and excluded from metric estimation. Findings should be interpreted as preliminary and hypothesis-generating, with replication in prospective, adequately powered designs needed before conclusions can be drawn about the directionality or magnitude of these relationships.

Engagement profiles were derived by applying median splits to the α values and Gini coefficients [23]. Median splits were used to facilitate interpretation and generate meaningful, mutually exclusive engagement profiles based on the intersection of interaction depth and temporal consistency. This approach was selected for its interpretive utility and aligns with the study’s aim of identifying distinct, real-world engagement patterns. It is also consistent with prior work in digital health that has used similar categorization strategies to characterize user engagement [7,8]. Alternative classification strategies, such as cluster analysis or tertile splits, were considered but not pursued, given the exploratory nature of the study and the modest sample size. Users were first classified as extended (lower α values) or brief (higher α values). Separately, users were classified as consistent (lower Gini coefficients) or episodic (higher Gini coefficients). These classifications were then cross-tabulated to create 4 mutually exclusive groups defined by combinations of interaction depth (extended versus brief) and temporal consistency (episodic versus consistent). To ensure reliable estimates, Gini and α values were calculated only for users with ≥5 sessions [24]. This threshold was selected to ensure stable parameter estimation for both metrics, consistent with recommendations for fitting distributional models to small samples [20]. However, this threshold has not been empirically validated specifically for DMHI engagement data and should be interpreted with caution. A sensitivity analysis comparing engagement profiles and symptom associations under a ≥2-session threshold was conducted to empirically evaluate the stability and justification of the ≥5-session analytical criterion. Descriptive statistics for depression and anxiety were summarized across session count groups (1 session, 2‐4 sessions, and ≥5 sessions) for all users with available survey data. Correlations between engagement pattern metrics (α and Gini) and total duration were modest (|r|≤0.26), suggesting that pattern-based metrics capture distinct aspects of engagement.

Demographic and engagement characteristics were summarized using descriptive statistics, including mean and SD for continuous variables and total counts and percentages for categorical variables. Group differences were tested using one-way ANOVA for continuous variables (eg, age) and chi-square tests for categorical variables. A sensitivity analysis was conducted to compare included users in the analytic sample with excluded users based on demographic characteristics. Independent-samples *t* tests were used for continuous variables, and chi-square tests of independence were used for categorical variables. Means and SDs for depression and anxiety were calculated across engagement profiles. To compare differences in depression and anxiety across engagement profiles, one-way ANOVA was used. Post hoc comparisons were conducted using Tukey honestly significant difference test, and effect sizes were estimated using eta squared (*η*²).

Linear regression models were used to examine associations between engagement profiles, depression, and anxiety. Separate models were estimated for depression and anxiety as outcomes. The engagement profiles were included as a categorical predictor with the extended and episodic group as the reference category. The extended and episodic group was selected as the reference group to facilitate interpretation, as this profile exhibited the lowest levels of depression and anxiety in descriptive analyses. A sensitivity analysis was conducted without the adjustment for total duration of use, given that duration represents a component of engagement behavior. All models were adjusted for total duration of use, age, and gender. Missing data were handled using list wise deletion. Unstandardized regression coefficients (β), SEs, and *P* values are reported. Although Shapiro-Wilk tests indicated statistically significant departures from normality in model residuals (GAD-7: W=0.961, *P*=.002; PHQ-8: W=0.965, *P*=.005), skewness and kurtosis values were within acceptable ranges for linear regression with this sample size, and visual inspection of residual plots confirmed no severe distributional violations. Post hoc power analyses were conducted to evaluate the adequacy of the analytic sample for the primary ANOVA comparisons and regression models. Variance inflation factors were examined to assess multicollinearity among predictors and were all below 2, indicating no evidence of problematic collinearity. All analyses were conducted in R (version 4.3; R Foundation for Statistical Computing).

### Methods for the Mitigation of Bias

To mitigate potential conflicts of interest, the following safeguards were applied: (1) research questions were developed by the Fit Minded scientific team based on scientific merit and were not prescribed by the client; (2) study outcomes were not guaranteed as contractual deliverables; (3) the analysis plan was prespecified before data access; (4) data analysis and interpretation were led by Fit Minded authors operating independently of The Path; and (5) sensitivity analyses were conducted to evaluate the robustness of findings to alternative model specifications, including a comparison of ≥2 versus ≥5 session thresholds to evaluate the robustness of the engagement profile solution and symptom associations to the analytical inclusion criterion, and a model specification excluding total duration of use to evaluate the robustness of regression findings to this covariate. No author’s employment status or compensation is contingent upon the direction or outcome of the findings.

## Results

### User Characteristics and Engagement Profiles

A total of 209 Mental app users completed the survey. After removing duplicates, 205 distinct users were retained and successfully linked to back-end app usage data. Of these, 93 had fewer than 5 sessions and were excluded to ensure stable estimation of engagement metrics, yielding an analytic sample of 112 users. A sensitivity analysis comparing included and excluded users revealed that included users were older and more likely to have higher educational attainment. No significant differences in gender, ethnicity, or race were identified (see Multimedia Appendix 1).

Four engagement profiles were derived based on combinations of interaction depth and temporal consistency (Table 1). Profile 1 (extended and episodic; n=25) reflected deep but temporally clustered use; profile 2 (extended and consistent; n=31) reflected deep, regularly spaced use; profile 3 (brief and episodic; n=31) reflected short, clustered interactions; and profile 4 (brief and consistent; n=25) reflected brief but consistently spaced use. Across the analytic sample, participants were predominantly men (87/112, 77.68%), White (85/112, 75.89%), and non-Hispanic or Latinx (103/112, 91.96%), with a mean age of 39.6 (SD 8.1) years. Demographic characteristics did not significantly differ across engagement profiles (Table 2; all *P*>.05).

**Figure 1.**
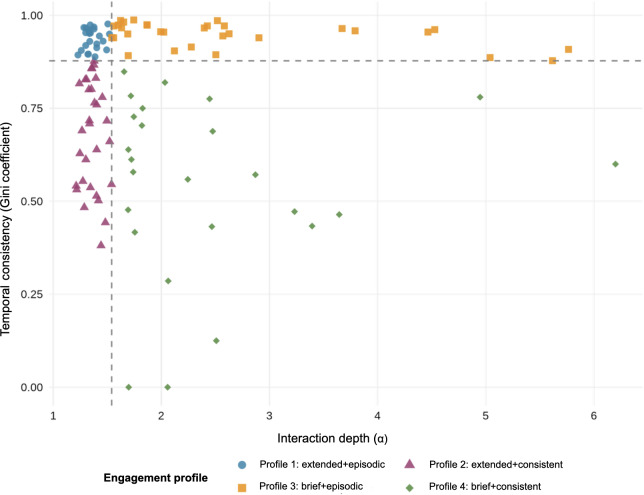
Engagement profile distribution by interaction depth and temporal consistency.

Engagement characteristics differed significantly across profiles (all *P*<.001; Table 1 ; Figure 1). Profile 2 (extended and consistent) demonstrated the highest mean number of sessions (mean 63.9, SD 78.35) and the greatest total duration of use (mean 1022.0, SD 1205.0 min), indicating sustained and regularly distributed engagement over time. Despite this overall pattern, substantial within-profile variability suggested considerable heterogeneity in cumulative app exposure among users with consistent engagement patterns. In contrast, profiles 3 and 4 averaged fewer than 13 sessions and under 181 total minutes of use, reflecting comparatively brief engagement overall. Profile 1 (extended and episodic) showed moderate overall use (mean 29.9, SD 16.39 sessions; mean 367.0, SD 316.45 min) but high temporal clustering, consistent with concentrated bursts of sustained interaction.

**Table 1.** Engagement characteristics and patterns by engagement profile based on interaction depth and temporal consistency (N=112).

Engagement characteristic	Profile 1^a^ (n=25), mean (SD)	Profile 2^b^ (n=31), mean (SD)	Profile 3^c^ (n=31), mean (SD)	Profile 4^d^ (n=25), mean (SD)	*P* value
Interaction depth^e^	1.36 (0.07)	1.36 (0.09)	2.64 (1.25)	2.47 (1.11)	<.001
Temporal consistency^f^	0.94 (0.03)	0.68 (0.14)	0.95 (0.03)	0.54 (0.24)	<.001
Total number of sessions	29.9 (16.39)	63.9 (78.35)	11.6 (7.91)	12.8 (13.01)	<.001
Total duration of use	367.0 (316.45)	1022.0 (1205.0)	181.00 (133.42)	172.00 (162.54)	<.001

a Extended and episodic group.

b Extended and consistent group.

c Brief and episodic group.

d Brief and consistent group.

e Lower α values indicate longer, more sustained sessions. Higher α values reflect brief interactions.

f Lower Gini coefficients indicate more consistent engagement over time. Higher Gini coefficients reflect temporally clustered, episodic usage patterns.

**Table 2.** User demographics by engagement profile (N=112)^a^.

Characteristic	Profile 1^b^ (n=25)	Profile 2^c^ (n=31)	Profile 3^d^ (n=31)	Profile 4^e^ (n=25)
Age (y), mean (SD)	36.96 (8.44)	41.48 (7.78)	40.39 (8.32)	39.28 (7.51)
Gender, n (%)				
Man	22 (88)	23 (74.19)	23 (74.19)	19 (76)
Other or nonbinary	1 (4)	—^f^	—	1 (4)
Woman	2 (8)	8 (25.81)	8 (25.81)	5 (20)
Ethnicity, n (%)				
Hispanic or Latinx	2 (8)	4 (12.90)	1 (3.23)	2 (8)
Not Hispanic or Latinx	23 (92)	27 (87.09)	30 (96.77)	23 (92)
Race, n (%)				
Asian	2 (8)	1 (3.23)	3 (9.68)	1 (4)
Black	2 (8)	—	2 (6.45)	2 (8)
Multiracial	1 (4)	5 (16.13)	3 (9.68)	—
Other	1 (4)	3 (9.68)	—	1 (4)
White	19 (76)	22 (70.97)	23 (74.19)	21 (84)
Education, n (%)				
Bachelor’s	8 (32)	8 (25.81)	12 (38.71)	6 (24)
Graduate	8 (32)	7 (22.58)	9 (29.03)	7 (28)
High school or less	3 (12)	3 (9.68)	1 (3.23)	4 (16)
Some college or associate	6 (24)	12 (38.71)	9 (29.03)	8 (32)

a Demographic characteristics did not significantly differ across engagement profiles (all *P*>.05).

b Extended and episodic group.

c Extended and consistent group.

d Brief and episodic group.

e Brief and consistent group.

f Not applicable.

### Depression and Anxiety Characteristics Across Engagement Profiles

Descriptive symptom data were summarized across session count groups (ie, 1 session, 2‐4 sessions, and ≥5 sessions). Users with only 1 session (n=13) reported the highest mean depression (mean 12.08, SD 6.69) and anxiety (mean 10.38, SD 6.93) of any group. Users with 2 to 4 sessions (n=77) reported intermediate symptom levels comparable to the analytic sample. Depression differed significantly across session count groups (*F*_2,199_=3.12, *P*=.046), with the analytic sample reporting significantly lower depression than the 1-session group (mean difference=4.93, *P*=.04); anxiety differences were not significant (*F*_2,199_=2.40, *P*=.09). Full results are presented in Multimedia Appendix 1.

A one-way ANOVA revealed significant differences across engagement profiles (*F*_3,108_=5.47, *P*=.002, *η*²=.13). Post hoc comparisons indicated that users in profile 2 (extended and consistent; mean difference=4.81, *P*=.04) and profile 4 (brief and consistent; mean difference=7.08, *P*=.001) reported significantly higher depression than the extended and episodic group.

For anxiety, a one-way ANOVA revealed significant differences in anxiety across engagement profiles (*F*_3,108_=8.07, *P*<.001, *η*²=.18; Table 3). Overall, profiles characterized by shorter and more consistent use (particularly profile 4) showed the highest symptom levels, whereas the extended and episodic profile, marked by longer sessions occurring less frequently, showed the lowest. Post hoc Tukey tests indicated that profile 2 (extended and consistent; mean difference=5.29, *P*=.003) and profile 4 (brief and consistent; mean difference=7.36, *P*<.001) reported significantly higher anxiety than the extended and episodic profile. Profile 4 (brief and consistent) reported higher anxiety than profile 3 (brief and episodic; mean difference=4.07, *P*=.04).

**Table 3.** Depression and anxiety across engagement profiles (N=112).

Engagement profile	Depression, mean (SD)	Anxiety, mean (SD)
Profile 1^a^ (n=25)	3.48 (4.06)	2.68 (2.43)
Profile 2^b^ (n=31)	8.29 (6.53)	7.97 (6.32)
Profile 3^c^ (n=31)	6.23 (5.93)	5.97 (5.22)
Profile 4^d^ (n=25)	10.60 (8.75)	10.00 (7.03)

a Extended and episodic group.

b Extended and consistent group.

c Brief and episodic group.

d Brief and consistent group.

Post hoc power analyses indicated adequate statistical power across analyses. For the one-way ANOVA comparisons, power was 94% for depression (*η*²=0.13, *f*=0.39) and 99% for anxiety (*η*²=0.18, *f*=0.47), assuming 4 groups, α=.05, and the observed minimum group size (n=25).

### Associations Between Engagement Profiles and Mental Health Symptom Severity

Separate linear regression models were used to examine associations between engagement profiles and depression and anxiety symptom severity, adjusting for age, gender, and total duration of use. Engagement profile was significantly associated with depressive symptom severity, *F*_7,104_=2.87, *P*=.009 (Table 4). Overall, profiles characterized by more frequent or regular use were associated with higher depressive symptom severity relative to the extended and episodic reference group. Compared to profile 1 (extended and episodic), users in profile 2 (extended and consistent; β=5.16, *P*=.008) and profile 4 (brief and consistent; β=6.85, *P*<.001) reported significantly higher depressive symptom severity. Profile 3 (brief and episodic) did not significantly differ from the reference profile (ie, profile 1; extended and episodic; *P*=.14).

**Table 4.** Linear regression models examining associations between engagement profiles and mental health symptom severity.

Predictor^a^^,^^b^	β^c^ (SE)	*P* value
Depression^d^		
Profile 2^e^	5.16 (1.90)	.008
Profile 3^f^	2.67 (1.81)	.14
Profile 4^g^	6.85 (1.87)	<.001
Anxiety^h^		
Profile 2	5.28 (1.62)	.002
Profile 3	3.11 (1.54)	.047
Profile 4	7.11 (1.60)	<.001

a Extended and episodic group.

b The engagement profile was modeled as a categorical predictor with profile 1 as the reference group. All models were adjusted for age, gender, and total duration of use.

c Unstandardized regression coefficients.

d Model fit: Patient Health Questionnaire-8 model, *R*²=0.16, *F*_7,104_=2.87, and *P*=.009.

e Extended and consistent group.

f Brief and episodic group.

g Brief and consistent group.

h Model fit: Generalized Anxiety Disorder-7 model,﻿ *R*²=0.21, *F*_7,104_=4.04, and *P*<.001.

Engagement profile was significantly associated with anxiety, *F*_7,104_=4.04, *P*<.001. Similarly to depression, profiles characterized by more frequent or regular use were associated with higher anxiety symptom severity relative to the extended and episodic reference group. Relative to profile 1 (extended and episodic), users in profile 2 (extended and consistent; β=5.28, *P*=.002), profile 3 (brief and episodic; β=3.11, *P*=.047), and profile 4 (brief and consistent; β=7.11, *P*<.001) all reported significantly higher anxiety symptom severity. Sensitivity analyses excluding total duration of use from regression models yielded consistent results, with engagement profiles remaining significantly associated with both depression (profile 2: β=4.88, *P*=.008; profile 4: β=6.95, *P*<.001) and anxiety (profile 2: β=5.17, *P*=.001; profile 3: β=3.15, *P*=.04; profile 4: β=7.15, *P*<.001), supporting the robustness of findings to this model specification (see Multimedia Appendix 1). For the overall regression models, power was 93% for depression (*R*²=0.16, *f*²=0.19) and 99% for anxiety (*R*²=0.21, *f*²=0.27).

Findings were further supported by a session threshold sensitivity analysis, in which engagement profiles derived under a ≥2-session criterion yielded substantially attenuated effect sizes (GAD-7: *η*²=.02 and PHQ-8: *η*²=.03) and nonsignificant regression associations, indicating that the ≥5-session threshold produced more stable and discriminable engagement profiles and stronger associations with symptom severity (see Multimedia Appendix 1).

## Discussion

### Principal Findings

The purpose of this cross-sectional study was to explore how different patterns of engagement with the Mental conversational AI agent relate to self-reported depression and anxiety. We aimed to (1) identify and describe engagement profiles based on the patterns of interaction depth and temporal consistency, (2) compare depression and anxiety symptoms across engagement profiles, and (3) explore whether engagement profiles were associated with depression and anxiety symptom severity. Four distinct engagement profiles emerged (profiles 1 through 4), capturing variation in session depth and temporal distribution of use. Depression and anxiety symptoms significantly differed across profiles. Users in profile 1 (extended and episodic), who engaged in longer sessions across fewer days, reported the lowest symptom levels, while users in profile 4 (brief and consistent), who engaged in shorter sessions spread more evenly over time, reported the highest. More broadly, users whose engagement was more evenly distributed across days reported higher symptoms regardless of session depth. After adjusting for age, gender, and total duration of use, engagement profiles remained significantly associated with both depression and anxiety, with higher symptom severity associated with more consistent use and lower symptom severity associated with extended but episodic use. These findings suggest that pattern-based engagement metrics may capture clinically meaningful variation that volume-based measures alone cannot and generate testable hypotheses for prospective investigation.

To our knowledge, this is the first study to characterize patterns of engagement among users interacting with an AI conversational agent in the real world. Using α and Gini metrics, 4 distinct profiles emerged, reflecting combinations of session depth (ie, long vs short interactions) and temporal patterns of use (ie, evenly distributed vs clustered across days). Prior work in DMHIs has primarily characterized engagement using raw session counts or total time on the platform [25]. The α and Gini metrics used here offer 2 specific advantages over traditional volume-based measures. First, they characterize the shape and distribution of engagement behavior rather than its aggregate quantity, capturing variation that session counts and total time obscure [10]. Second, by quantifying both interaction depth and temporal consistency independently, they allow engagement to be decomposed into dimensions that map onto theoretically meaningful constructs from the psychotherapy dosing literature [13]. In doing so, these profiles move beyond a single engagement quantity toward a multidimensional characterization of how users interact with AI-powered care.

Users who engaged consistently over time, regardless of whether their individual sessions were long or short, reported higher depression and anxiety symptoms than those whose use was more temporally clustered. This pattern is consistent with prior research on digital health behavior, suggesting that individuals with greater symptom severity may seek out more regular platform engagement as a coping resource [26]. This interpretation is further supported by the on-demand nature of the platform, which removes structural barriers to access and may facilitate frequent, symptom-driven use among users with persistent or unmet support needs. Notably, users in profile 4 (brief and consistent) reported the highest symptom levels of any group, suggesting that frequent, brief interactions distributed over time may reflect an ongoing need for support rather than a pattern associated with symptom improvement. In contrast, users who engaged in longer, more in-depth sessions that were concentrated within fewer days (ie, extended and episodic use) reported the lowest symptom levels. These findings may carry implications for DMHI design. Specifically, the divergence between profile 1 (lowest symptoms; extended and episodic) and profile 4 (highest symptoms; brief and consistent) suggests that temporal clustering of sustained interactions may be a more relevant engagement signature than consistency alone. The association between consistent engagement and higher symptoms diverges from the psychotherapy literature, in which regular session attendance is generally associated with better outcomes [12,26]. This divergence may reflect a fundamental difference between human-delivered therapy and AI-powered therapy. In human-delivered therapy, consistency is clinician-scheduled and reflects treatment adherence, whereas in AI-powered care, it is entirely user-initiated and may instead reflect symptom burden or unmet need. This structural distinction has direct implications for how engagement data should be interpreted in AI-powered care and underscores why pattern-based metrics that capture both depth and timing are preferable to aggregate volume as indicators of meaningful use. Preliminary work suggests AI-powered interventions may produce self-reported improvement rates that are comparable to, or exceed, those observed in traditional outpatient settings across multiple well-being domains and often in fewer total hours of engagement [5,6]. Understanding how users engage, not just how much, will be essential for interpreting those comparisons meaningfully.

The adjusted regression models further demonstrated that engagement profiles were associated with depression and anxiety beyond total duration of use alone, providing preliminary evidence that pattern-based metrics (eg, temporal consistency and interaction depth) may offer explanatory value beyond volume-based measurement. This finding aligns with a growing recognition in the DMHI literature that simple volume metrics, such as total sessions or time on platform, are insufficient proxies for meaningful engagement [7,9]. This parallels findings from the psychotherapy dose-response literature, in which session frequency has been shown to be associated with treatment outcomes independent of the total number of sessions attended [11,27,28]. Taken together, the present findings provide preliminary empirical support for the value of pattern-based engagement measurement as a complement to, or replacement for, aggregate usage metrics in DMHI research and evaluation, pending replication in prospective designs.

The engagement profiling approach introduced here opens several avenues for future research. Prospective designs with pre-post symptom measurement, longitudinal follow-up, and control groups are needed to establish the directionality of the relationships observed here and to determine whether engagement profiles are associated with symptom change over time. Replication among more demographically diverse samples will be important for assessing the generalizability of these profiles beyond the predominantly White population of men examined here. Research should also explore whether real-time detection of engagement patterns can inform adaptive platform responses, such as prompts toward longer sessions for users exhibiting brief, consistent use. Extending this profiling framework to other DMHI modalities would help establish whether these patterns reflect stable features of user behavior or are specific to conversational AI. A particularly important next step is the systematic comparison of AI-powered and human-delivered outpatient care using standardized baseline measures, matched comparison groups, and pre-post validated outcome instruments, with engagement pattern metrics included as potential moderators of comparative effectiveness. Future research should also examine the sensitivity of engagement profile solutions to alternative classification strategies, including cluster-based approaches and varied session thresholds, to establish whether the profiles identified here represent stable behavioral phenotypes or are sensitive to analytical choices. Benchmark comparisons using established instruments such as the Mental Health Statistics Improvement Program (MHSIP) provide a useful starting point [29], but prospective, preregistered designs are needed to establish the conditions under which AI therapy is most effective relative to traditional care.

This study has several strengths. To our knowledge, this is the first application of α and Gini coefficient metrics to derive engagement profiles in a DMHI, offering a more nuanced approach to characterizing real-world user behavior than traditional volume-based metrics. The use of real-world app data linked to validated clinical measures (PHQ-8, GAD-7) strengthens ecological validity, and adjusted regression models accounted for key covariates (age, gender, and total duration of use), isolating profile-level effects on mental health symptom severity.

Several limitations should also be noted. First, this exploratory, cross-sectional observational design precludes causal inference, and the direction of the observed associations cannot be determined from these data. It is plausible that users with greater symptom severity engage with the platform more consistently as a coping response, just as it is plausible that engagement patterns themselves influence symptom levels, a reverse causality interpretation that cannot be ruled out. Although post hoc power analyses indicated adequate statistical power for the primary comparisons, statistical power does not address the fundamental constraints of the cross-sectional design, and prospective replication with pre-post measurement, larger and more diverse samples, and control conditions is necessary before conclusions about generalizability or clinical meaningfulness can be drawn. Second, survey respondents were self-selected to participate and may differ systematically from nonrespondents, potentially inflating or deflating self-reported symptom estimates. The incentivized design, in which participants received a US $20 gift card for survey completion, may have further introduced response bias by disproportionately attracting users with stronger opinions about the platform. Third, the sample (n=112) was modest and predominantly White and men, limiting generalizability to other demographic groups. The platform’s explicit marketing toward adult men likely contributed to this skew and should be considered when interpreting findings; results may not generalize to women, gender-diverse individuals, or clinical populations with more severe or complex presentations. The retrospective design precluded a priori power estimation, and observed effect sizes should be interpreted cautiously pending replication in larger, prospectively designed studies. Moreover, the substantial attrition from 205 to 112 users due to the ≥5-session inclusion threshold represents a 45.36% (n=93) reduction in the available sample and raises the possibility of systematic bias. Users with fewer sessions may represent a meaningfully different population in terms of engagement motivation, symptom severity, or platform experience, limiting the extent to which findings can be generalized to lower-engagement users. Additionally, a sensitivity analysis indicated that included users were older and more likely to have higher educational attainment than excluded users, suggesting that excluded users may represent a less engaged or earlier-stage user population. Older age and higher educational attainment among included users may reflect greater familiarity with digital health tools or higher motivation to sustain platform use, limiting generalizability to younger or less educated users who disengage from the platform earlier. Prospective studies with larger, more diverse samples are needed to address these limitations and establish the directionality and generalizability of these findings.

Fourth, the session threshold of ≥5 and the use of median splits represent pragmatic analytical choices that, while consistent with prior work [7,9,23], impose boundaries on continuous distributions and may not optimally capture the full range of engagement behavior in this sample. The attenuation of effects observed under the ≥2-session threshold is consistent with the theoretical requirements of distributional metrics: the Gini coefficient requires sufficient temporal observations to meaningfully characterize inequality in session spacing, and the Pareto-based α parameter requires sufficient session duration observations to estimate distributional shape. This pattern of attenuation suggests the threshold difference reflects a psychometric requirement rather than an arbitrary analytical choice, though the optimal minimum threshold for these metrics in DMHI contexts has not been empirically established. Fifth, the application of Pareto-based metrics to session duration data in DMHIs is a novel methodological approach. Given that session durations were drawn from a discrete set of platform-defined lengths, standard goodness-of-fit procedures were not fully appropriate, and distributional fit was evaluated through visual inspection of the log-log complementary cumulative distribution function, which confirmed approximately linear decay consistent with heavy-tailed distributional assumptions. Future work should formally validate α-based engagement metrics in DMHI populations, examine alternative distributional models, and systematically test the sensitivity of engagement profile solutions to varied session thresholds and classification strategies to establish whether the profiles identified here represent stable behavioral phenotypes.

### Conclusions

This study introduces a novel engagement profiling approach to characterize how users interact with an AI-powered mental health intervention, moving beyond traditional volume-based metrics to capture both session depth and temporal patterns of use. Four distinct profiles emerged, and engagement patterns were significantly associated with self-reported depression and anxiety after adjusting for total duration of use. Users who engaged in deeper, temporally clustered sessions reported the lowest symptom levels, while those with brief, evenly distributed use reported the highest. These findings suggest that not all engagement is equal, and that the structure of interaction may be as informative as its quantity. As conversational AI agents become increasingly integrated into mental health care delivery, understanding the relationship between engagement patterns and clinical outcomes will be essential for optimizing platform design and identifying users who may benefit from tailored support. These results are preliminary and hypothesis-generating; prospective designs with diverse samples are needed to establish directionality and generalizability.

## Supplementary material

10.2196/98690 Multimedia Appendix 1 Mental app impact survey, including the complete questionnaire and survey items administered to participants, and supplemental analyses.

10.2196/98690 Checklist 1 STROBE checklist.

10.2196/98690 Checklist 2 CHERRIES checklist.

## Abbreviations

BRANY: Biomedical Research Alliance of New York
CBT: cognitive behavioral therapy
CHERRIES: Checklist for Reporting Results of Internet E-Surveys
DMHI: digital mental health intervention
GAD-7: Generalized Anxiety Disorder-7
MHSIP: Mental Health Statistics Improvement Program
PHQ-8: Patient Health Questionnaire-8
STROBE: Strengthening the Reporting of Observational Studies in Epidemiology
