# Supplementary material for: Patterns of Engagement With an AI Conversational Agent for Mental Health and Associations With Anxiety and Depression: Cross-Sectional Study
Source: JMIR Ment Health. 2026 Aug 10;13:e98690. doi: 10.2196/98690 (PMC13456072; doi:10.2196/98690)
Supplement: Supplementary file 1 [file mental-v13-e98690-s001.docx]

**Patterns of Engagement With an AI Conversational Agent for Mental Health and Associations With Anxiety and Depression: Cross-Sectional Study**

**Supplement Material**

**Mental App Impact Survey**

Note: This survey was administered to users of both the Mental and Pathos apps, which share a common platform infrastructure developed by The Path. The present study includes only Mental app users. References to 'Mental and/or Pathos' throughout the survey reflect the original instrument as administered.

**Demographics**

1. What is your age (in years)?
2. What is your current gender (check all that apply)?

- Man
- Woman
- Transgender Man/Transman
- Transgender Woman/Transwoman
- Genderqueer
- Additional Category: ______________
- Decline to State

3. What is your race?

- White, European-American, or Caucasian
- Black, African American, or Native African
- Asian or Asian-American
- American Indian or Alaskan Native
- Native Hawaiian or Pacific Islander
- Middle Eastern/North African (MENA)
- Bi-racial or Multi-racial
- Other (please specify)

4. Do you identify as Hispanic or Latinx?

- Yes
- No

5. What is your highest level of education?

- 11th grade or less
- High school diploma or GED
- Some college
- Two-year college/technical school degree
- Bachelor's degree or equivalent (e.g., BA, BS)
- Graduate degree or equivalent (MA, MD, PhD)
- Other: ___

**Mental/Pathos Use**

6. What is the main reason you started using Mental and/or Pathos?

- I was struggling with my mental health
- I wanted help in the moment
- I wanted a more private way to get support
- I was curious about AI-based support
- Other (please specify)

7. How often do you use Mental and/or Pathos?

- Multiple times per day
- Once per day
- 5–6 times per week
- 3–4 times per week
- 1–2 times per week
- 2–3 times per month
- About once per month
- Less than once per month
- I no longer use the Mental/Pathos app

8. IF NO LONGER USING MENTAL/PATHOS: When you were using Mental and/or Pathos, how often did you use it?

- Multiple times per day
- Once per day
- 5–6 times per week
- 3–4 times per week
- 1–2 times per week
- 2–3 times per month
- About once per month
- Less than once per month

9. IF NO LONGER USING MENTAL/PATHOS: What is the main reason you stopped using Mental and/or Pathos?

- I felt better and no longer needed support
- I found another form of support that worked better for me
- I didn’t find Mental/Pathos helpful enough
- I didn’t have time or forgot to use it
- I had concerns about using an AI for support
- I stopped because of a technical or access issue
- Other (please specify)

**Impact on Mental Health**

Take a moment to think about your experience with Mental and/or Pathos. For each of the areas below, let us know how things have changed for you since you started using the app. *Rating scale: 1=much worse, 2=worse, 3=no change, 4=better, 5=much better, not applicable*

Since using Mental/Pathos, how have the following changed for you?

10. Anxiety or worry
11. Sadness or low mood
12. Loneliness or social disconnection
13. Stress level
14. Irritability or frustration
15. Being overwhelmed
16. Low motivation or low energy
17. Sleep difficulties
18. Difficulty coping with strong or difficult emotions
19. Overall emotional distress

Since using Mental/Pathos, how have the following changed for you?

20. Sense of purpose or direction
21. Confidence in handling daily challenges
22. Self-awareness and emotional insight
23. Hopefulness about the future
24. Satisfaction with life overall

**Impact on Work Productivity**

Take a moment to think about your experience with Mental and/or Pathos. For each of the areas below, let us know how things have changed for you since you started using the app. *Rating scale: 1=much worse, 2=worse, 3=no change, 4=better, 5=much better, not applicable*

Since using Mental/Pathos, how have the following changed for you?

25. Feelings of burnout at work
26. Overall work productivity
27. Ability to stay focused at work
28. Ability to manage work-related stress
29. Attendance at work (e.g., missed days)

**Impact on Healthcare Use**

Take a moment to think about your experience with Mental and/or Pathos. For each of the areas below, let us know how things have changed for you since you started using the app. *Rating scale: 1=much worse, 2=worse, 3=no change, 4=better, 5=much better, not applicable*

Since using Mental/Pathos, how have the following changed for you?

30. Use of emergency or crisis mental health services
31. Use of hospital-based mental health care
32. Unplanned medical visits related to mental health
33. Need for immediate professional mental health intervention

**AI Safety and Trust**

Please indicate how much you agree or disagree with the following statements about your experience using the AI therapist. *Rating scale: 1=strongly disagree, 2=disagree, 3=neither agree nor disagree, 4=agree, 5=strongly agree*

34. I feel safe interacting with the AI therapist.
35. The AI therapist handles sensitive topics carefully.
36. The AI therapist communicates clearly.
37. I feel comfortable sharing personal information with the AI therapist.
38. I understand the limitations of the AI therapist.

**Trust in Automation Scale Short Form (**[**S-TIAS**](https://pmc.ncbi.nlm.nih.gov/articles/PMC12098057/)**)**

*Response options: 1 = Not at all and 7 = Extremely*

39. I am confident in the AI therapist
40. The AI therapist is reliable
41. I can trust the AI therapist

**Comparison to Competitors**

42. Have you used any other forms of mental health or emotional support?

- Yes
- No

43. IF YES: Which of the following have you used? (Select all that apply)

- In-person therapy or counseling
- Online therapy (video or messaging)
- Psychiatry or medication management
- Primary care support for mental health
- Mental health coaching
- Support groups (in-person or online)
- Crisis hotline or emergency support
- Self-guided mental health apps
- Meditation or mindfulness apps
- Other (please specify)

IF YES: Please think about other forms of support you’ve used in the past (e.g., therapy, coaching, other mental health apps). Indicate how much you agree or disagree with the following statements. *Rating scale: 1=strongly disagree, 2=disagree, 3=neither agree nor disagree, 4=agree, 5=strongly agree.*

Compared to other forms of support I’ve used:

44. Mental/Pathos is easier to access when I need support.
45. Mental/Pathos feels more personalized to my needs.
46. Mental/Pathos is more helpful in the moment.
47. I feel more comfortable using Mental/Pathos.
48. Mental/Pathos has improved my mental health more than other options.
49. I feel less judged using Mental/Pathos than other forms of support.

**Validated Measures**

**Depression (PHQ-8)**

Instructions: Over the last 2 weeks, how often have you been bothered by any of the following problems? *Response options: 0=Not at all, 1=Several days, 2=More than half the days, 3=Nearly every day*

1. Little interest or pleasure in doing things
2. Feeling down, depressed, or hopeless
3. Trouble falling or staying asleep, or sleeping too much
4. Feeling tired or having little energy
5. Poor appetite or overeating
6. Feeling bad about yourself or that you are a failure or have let yourself or your family down
7. Trouble concentrating on things, such as reading the newspaper or watching television
8. Moving or speaking so slowly that other people could have noticed. Or the opposite being so fidgety or restless that you have been moving around a lot more than usual

**Anxiety (GAD-7)**

Instructions: Over the last 2 weeks, how often have you been bothered by the following problems? *Response options: 0=Not at all, 1=Several days, 2=More than half the days, 3=Nearly every day*

1. Feeling nervous, anxious, or on edge
2. Not being able to stop or control worrying
3. Worrying too much about different things
4. Trouble relaxing
5. Being so restless that it's hard to sit still
6. Becoming easily annoyed or irritable
7. Feeling afraid as if something awful might happen

**Loneliness** ([UCLA 3 item](https://journals.sagepub.com/doi/pdf/10.1177/0164027504268574?casa_token=Ku6Wy5DXvgQAAAAA:DlH4t_66X5N39JU90i_RF1k5LJy1868DtJxp0jel8P3zK6Bsz6wCE5FnMQm7cB5tGYpCdGDkaMRM))

1. How often do you feel that you lack companionship:

- Hardly ever
- Some of the time
- Often

2. How often do you feel left out:
   - Hardly ever
   - Some of the time
   - Often
3. How often do you feel isolated from others?
   - Hardly ever
   - Some of the time
   - Often

**General Well-Being (WHO-5)**

Please indicate for each of the five statements which is closest to how you have been feeling over the last two weeks. *Response scale: 0 = At no time, 1 = Some of the time, 2 = Less than half of the time, 3 = More than half of the time, 4 = Most of the time, 5 = All of the time*

1. I have felt cheerful and in good spirits
2. I have felt calm and relaxed
3. I have felt active and vigorous
4. I woke up feeling fresh and rested
5. My daily life has been filled with things that interest me

**Therapeutic Alliance with Mental/Pathos (**[**DWAI**](https://pmc.ncbi.nlm.nih.gov/articles/PMC8599525/)**)**

*Response options: 1=strongly agree to 7=strongly disagree).*

1. I believe the app tasks will help me to address my problem
2. The app is easy to use and operate
3. I trust the app to guide me towards my personal goals
4. I agree that the tasks within the app are important for my goals
5. The app encourages me to accomplish tasks and make progress
6. The app supports me to overcome challenges

**Sensitivity analysis comparing included versus excluded participants.**

A sensitivity analysis was conducted to compare demographic characteristics between participants included in the analytic sample (n = 112) and those excluded (n = 93). Included participants were significantly older than excluded participants (M = 39.7, SD = 8.09 vs M = 36.2, SD = 9.31; p = .005). There were no statistically significant differences in gender distribution (p = .062), ethnicity (p = .163), or race (p = .990) between groups. See Table S3.

Educational attainment differed significantly (p = .030), with included participants more likely to have completed a bachelor’s or graduate degree and excluded participants more likely to report a high school education or less.

| Sensitivity Analysis comparing included versus excluded participants. | | | | | |
| --- | --- | --- | --- | --- | --- |
|  | Included  (n = 112) | | Excluded  (n = 93) | | P-value |
|  | M | SD | M | SD |  |
| Age | 39.7 | 8.09 | 36.2 | 9.31 | .005 |
|  | n | % | n | % |  |
| Gender | | | | | |
| Men | 87 | 77.7 | 59 | 63.4 | .062 |
| Women | 23 | 20.5 | 29 | 31.2 |  |
| Other/Non-binary | 2 | 1.8 | 5 | 5.4 |  |
| Ethnicity | | | | | |
| Hispanic/Latinx | 9 | 8.0 | 13 | 14.1 | .163 |
| Not Hispanic/Latinx | 103 | 92.0 | 80 | 85.9 |  |
| Race | | | | | |
| White | 85 | 75.9 | 70 | 75.3 | .990 |
| Asian | 7 | 6.3 | 7 | 7.5 |  |
| Black | 6 | 5.4 | 4 | 4.3 |  |
| Multiracial | 9 | 8.0 | 7 | 7.5 |  |
| Other | 5 | 4.5 | 5 | 5.4 |  |
| Education | | | | | |
| Bachelor’s | 34 | 30.6 | 23 | 25.3 | .030 |
| Graduate | 31 | 27.9 | 18 | 19.8 |  |
| Some college/Associate | 35 | 31.5 | 27 | 29.7 |  |
| High school or less | 11 | 9.9 | 23 | 25.3 |  |
| Note. | | | | | |

**Symptom Characteristics by Session Count Group.**

Descriptive statistics for depression (PHQ-8) and anxiety (GAD-7) are presented across session count groups. Users with only one session or two to four sessions were excluded from primary analyses, as the alpha and Gini metrics require a minimum of five sessions for stable estimation (Table S4). A one-way ANOVA indicated no significant differences in anxiety across groups (F(2, 199) = 2.40, p = .093). Depression differed significantly across groups (F(2, 199) = 3.12, p = .046); post hoc Tukey comparisons indicated that users in the ≥5-session analytic sample reported significantly lower depression than the 1-session group (mean difference = 4.93, p = .035). No significant differences were observed between the 2-4 session and ≥5-session groups for either outcome (PHQ-8: p = .884; GAD-7: p=.991), suggesting that symptom levels stabilize after the first session.

| Depression and Anxiety by Session Count Group. | | | |
| --- | --- | --- | --- |
|  | n | Depression  PHQ-8 | Anxiety  GAD-7 |
| Session Group |  | M (SD) | M (SD) |
| 1 session | 13 | 12.08 (6.69) | 10.38 (6.93) |
| 2-4 sessions | 77 | 7.62 (6.51) | 6.58 (5.59) |
| ≥5 sessions (analytic sample) | 112 | 7.15 (6.89) | 6.70 (6.07) |

**Sensitivity analysis examining associations between engagement profiles and mental health symptom severity without adjustment for total duration of use.**

A sensitivity analysis was conducted to examine associations between engagement profiles and mental health symptom severity without adjusting for total duration of app use (Table S5). For depression (PHQ-8), participants in Profile 2 (extended and consistent; B = 4.88, SE = 1.80, p = .008) and Profile 4 (brief and consistent; B = 6.95, SE = 1.86, p < .001) reported significantly higher symptom severity, while Profile 3 (brief and episodic) was not significantly different from the reference group (p = .125). For anxiety (GAD-7), all non-reference profiles were associated with significantly higher symptom severity, including Profile 2 (extended and consistent; B = 5.17, SE = 1.54, p = .001), Profile 3 (brief and episodic; B = 3.15, SE = 1.53, p = .042), and Profile 4 (brief and consistent; B = 7.15, SE = 1.58, p < .001).

| Sensitivity analysis examining associations between engagement profiles and mental health symptom severity without adjustment for total duration of use. | | | | |
| --- | --- | --- | --- | --- |
| Outcome | Predictor | B | SE | p-value |
| Depression (PHQ-8) | Profile 2 | 4.88 | 1.80 | .008 |
|  | Profile 3 | 2.77 | 1.79 | .125 |
|  | Profile 4 | 6.95 | 1.86 | <.001 |
| Anxiety (GAD-7) | Profile 2 | 5.17 | 1.54 | .001 |
|  | Profile 3 | 3.15 | 1.53 | .042 |
|  | Profile 4 | 7.15 | 1.58 | <.001 |
| Note. Profile 1 = extended and episodic; Profile 2 = extended and consistent; Profile 3 = brief and episodic; Profile 4 = brief and consistent. The engagement profile was modeled as a categorical predictor with *Profile 1* as the reference group. Models were adjusted for age and gender. | | | | |

**Comparisons of Primary (≥5 Sessions) and Sensitivity (≥2 Sessions) Engagement Thresholds.**

Sensitivity analyses compared the primary ≥5-session threshold (n = 112) with a broader ≥2-session threshold (n = 189). All 112 users in the primary analytic sample were retained in the ≥2-session sensitivity sample. Of these, 76 (67.9%) retained the same engagement profile classification across thresholds. Profile distributions differed between thresholds. Under the ≥5-session criterion, engagement profiles were distributed as follows: extended and episodic (n = 25, 22.3%), extended and consistent (n = 20, 17.9%), brief and episodic (n = 19, 17.0%), and brief and consistent (n = 12, 10.7%). Under the ≥2-session criterion, profiles were distributed as follows: extended and episodic (n = 55, 29.1%), extended and consistent (n = 40, 21.2%), brief and episodic (n = 39, 20.6%), and brief and consistent (n = 55, 29.1%).

The ≥2-session sensitivity analysis produced substantially smaller effect sizes for both GAD-7 (η² = .02) and PHQ-8 (η² = .03), and engagement profiles were no longer significantly associated with anxiety or depressive symptom severity in adjusted regression models. Although directional patterns of coefficients were partially consistent with primary analyses for some profiles, estimates were substantially attenuated and no longer discriminable across profiles, suggesting that sparse session data introduce substantial instability into both metric estimation and profile classification. These findings provide empirical support for the ≥5-session threshold, indicating that alpha- and Gini-derived engagement metrics require a minimum degree of repeated behavioral observation to produce stable and clinically meaningful engagement classifications. Accordingly, the ≥5-session threshold was retained as the primary analytic specification given its superior stability and interpretive utility relative to the ≥2-session alternative.

| Adjusted Linear Regression Models Comparing Primary (≥5 Sessions) and Sensitivity (≥2 Sessions) Engagement Thresholds. | | |
| --- | --- | --- |
| Depression | | |
| Predictor | ≥5 Sessions B (p) | ≥2 Sessions B (p) |
| Profile 2 | 5.16 (.008) | 2.01 (.148) |
| Profile 3 | 2.65 (.145) | -0.23 (.872) |
| Profile 4 | 6.82 (<.001) | -1.12 (.381) |
| Anxiety | | |
| Profile 2 | 5.28 (.002) | 2.05 (.093) |
| Profile 3 | 3.11 (.047) | -0.44 (.720) |
| Profile 4 | 7.11 (<.001) | -0.24 (.830) |
| Note. Profile 1 = extended and episodic; Profile 2 = extended and consistent; Profile 3 = brief and episodic; Profile 4 = brief and consistent. The engagement profile was modeled as a categorical predictor with Profile 1 as the reference group.  R² = proportion of variance explained by the full regression model; adjusted R² adjusts for model complexity and sample size. Models adjusted for age, gender, and total duration of app use. Primary analyses included users with ≥5 sessions (n = 112), whereas sensitivity analyses included users with ≥2 sessions (n = 189). Model explanatory power was substantially reduced under the ≥2-session threshold. | | |
